# Time-dependent biphasic alterations in brain metabolism following chronic ketamine exposure in mice

**DOI:** 10.3389/fphar.2025.1629824

**Published:** 2025-11-14

**Authors:** Seo-Hyun Lim, Young-Suk Choi, Eosu Kim, Chul Hoon Kim, Ho-Taek Song

**Affiliations:** 1 Department of Radiology and Research Institute of Radiological Science, Yonsei University College of Medicine, Seoul, Republic of Korea; 2 Department of Psychiatry, Institute of Behavioral Science in Medicine, Yonsei University College of Medicine, Seoul, Republic of Korea; 3 Department of Pharmacology, Yonsei University College of Medicine, Seoul, Republic of Korea; 4 BK21 PLUS Project for Medical Sciences and Brain Research Institute, Yonsei University College of Medicine, Seoul, Republic of Korea

**Keywords:** ketamine, FDG-PET, brain metabolism, GLUT1, chronic exposure

## Abstract

**Background:**

Ketamine has attracted clinical interest for its therapeutic potential, but prolonged exposure raises concerns about dependence and its long-term effects on brain metabolism.

**Materials and Methods:**

Male mice received daily intraperitoneal injections of ketamine (30 mg/kg) for 28 days. Brain glucose metabolism was evaluated using [^18^F]FDG positron emission tomography at 1 h, 1 week, and 1 month post-injection. Expression levels of glucose transporters (GLUT1), glycolytic enzymes (PKM2, HK1), NMDA receptor subunits (NR2B), and apoptotic markers (caspase-3) were analyzed by Western blotting and RT-PCR.

**Results:**

FDG-PET imaging suggested a biphasic metabolic pattern, with an increase in uptake at 1 h and 1 week, followed by a significant reduction by 1 month, returning toward baseline levels. GLUT1 mRNA expression gradually increased, although protein levels did not show a clear parallel change. PKM2 and HK1 remained largely unchanged. At 1 month, NR2B and caspase-3 transcripts were elevated, while protein-level changes were less evident, suggesting possible transcriptional regulation of stress-related pathways.

**Discussion:**

These findings demonstrate that ketamine induces dynamic alterations in brain glucose metabolism accompanied by molecular adaptations. The early hypermetabolic response may reflect acute excitatory effects, whereas longer exposure could engage compensatory or stress-associated mechanisms. Metabolic imaging may provide a useful, non-invasive approach to better understand ketamine’s temporal effects and support long-term safety monitoring.

## Introduction

Ketamine is a widely used anesthetic and NMDA (N-Methyl-D-Aspartate) receptor antagonist that has gained attention for its rapid antidepressant effects, particularly in treatment-resistant depression. However, in the context of repeated or prolonged administration, concerns about its long-term neurobiological consequences, especially addiction and metabolic disturbances, are growing (Alnefeesi et al., 2022; Strous et al., 2022). Chronic ketamine use alters normal reward circuitry and induces neuroplastic changes that reinforce addictive behaviors (Mkrtchian et al., 2021). While traditional addiction research has primarily focused on neurotransmitter dynamics, the broader metabolic effects of prolonged ketamine exposure remain poorly understood (Datta et al., 2023). Given the established link between metabolic dysregulation and psychiatric disorders, investigating how ketamine influences brain metabolism is critical to understanding its maladaptive consequences under chronic exposure, distinct from its short-term therapeutic effects.

Ketamine has been reported to affect brain energy metabolism, including changes in ATP levels and glucose utilization, which are believed to contribute to its neuroplasticity-related effects (Davis et al., 1988; Weckmann et al., 2017). These alterations involve pathways such as glycolysis and oxidative phosphorylation, yet their long-term implications in the setting of chronic, repeated exposure remain unclear.

Metabolic imaging after ketamine exposure provides critical insights into the temporal dynamics of its effects on neural activity and energy metabolism. [^18^F]fluorodeoxyglucose positron emission tomography (FDG-PET) is a highly sensitive tool for detecting metabolic alterations in brain regions involved in addiction (Car et al., 2013). While prior studies have primarily reported increased glucose metabolism following ketamine administration, region- and time-dependent variations have been observed, highlighting the need to systematically investigate metabolic shifts associated with prolonged exposure and potential abuse (Ouyang et al., 2021). Given ketamine’s dual therapeutic and addictive potential, understanding the long-term metabolic consequences of chronic exposure is essential to distinguish its pharmacological effects from maladaptive neurobiological adaptations. However, the relationship between FDG-PET findings and molecular changes remains poorly understood. Elucidating these metabolic adaptations is critical for developing a comprehensive understanding of ketamine-induced neurobiological alterations in the context of chronic use and abuse liability.

To address these gaps, this study investigates the temporal dynamics of brain energy metabolism following ketamine exposure using FDG-PET imaging. We also examine molecular changes, focusing on glycolysis-related gene expression, caspase-3 as a marker of neuronal apoptosis, and the expression of NMDA and GABA (Gamma-Aminogutyric Acid) receptors. Although ketamine is known to induce widespread metabolic alterations across the brain, previous studies have highlighted the striatum as a critical region involved in its neuropharmacological effects, particularly in relation to dopamine regulation and reward processing (Mkrtchian et al., 2021; Chen et al., 2020). Therefore, this study aimed to correlate whole-brain metabolic changes observed by FDG-PET with molecular changes specifically in the striatum.

Our results show that while ketamine administration initially increases brain glucose uptake, prolonged ketamine exposure leads to return to baseline glucose metabolism levels. This normalization is accompanied by increased caspase-3 expression and upregulation of a NMDA receptor subunit, suggesting that sustained ketamine exposure may trigger neuroadaptive processes that reflect maladaptive outcomes associated with chronic use and addiction risk, rather than antidepressant responses.

## Methods

### Animal preparation

ICR male mice (4 weeks old, 25–30 g) were used in this study. All animal procedures were approved by the Yonsei University Laboratory Animal Research Center (YLARC; Approval No. 2024-0103) and conducted in accordance with NIH guidelines. The animals were housed in a specific pathogen-free facility at YLARC under controlled conditions, maintaining a stable temperature of 23 °C, with a 12-h light and 12-h dark cycle and free access to food and water. Two separate groups of mice (n = 3 per group) were used for tissue harvesting and longitudinal FDG-PET imaging, respectively. For molecular analyses, brain tissue was selectively collected from the striatum, a region highly responsive to ketamine-induced neurobiological changes (Chen et al., 2020). In the imaging group, the same animals were repeatedly scanned over the study period and subsequently euthanized for further analysis. This design allowed for the assessment of both metabolic and molecular alterations in the striatum, a core structure involved in motivation and reward processing, and known to be affected by ketamine exposure.

**FIGURE 1:**
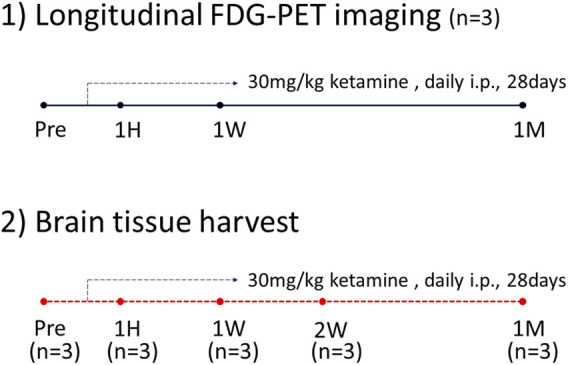
FDG-PET timeline.

To distinguish between the tissue harvesting and FDG-PET imaging groups, we set up two separate groups, each consisting of 3 mice (n = 3 per group). For tissue harvesting, mice were euthanized at four time points (pre, 1H, 1W, and 1M), with brain tissue collected for RNA isolation and histochemical analysis. For FDG-PET imaging, the same three mice were used at all-time points (pre, 1H, 1W, and 1M) over the course of a month, with imaging conducted prior to euthanasia at each designated time point. The mice were then humanely euthanized after imaging for further analysis (Figure 1).

### Ketamine administration method

Ketamine used in this study was obtained from Tocris Bioscience (Cat# KETA003) as a racemic mixture containing both R- and S-enantiomers. Mice received daily intraperitoneal injections of ketamine (30 mg/kg) for 28 days, and the injected volume was adjusted according to body weight. Because the purpose of this study was to investigate the consequences of sustained ketamine exposure leading to dependence, the dosing regimen was based on a previously established rodent model of ketamine addiction (Ding et al., 2016).

### FDG-PET imaging

All PET scans were performed with the preclinical PET scanner Inveon (Siemens Medical Solutions, Knoxville, United States) small animal scanner at the ABMRC Imaging Center, Yonsei University. Male ICR mice (n = 3) performed FDG imaging at four time points: before treatment (Pre), and at 1 h (1H), 1 week (1W), and 4 weeks (4W) after initiating daily ketamine (30 mg/kg, intraperitoneal injection) injections for 28 days. In all experiments, only male mice were used to minimize potential confounding effects, as hormonal fluctuations during the estrous cycle in female mice can influence metabolic and molecular outcomes. All mice had unrestricted access to food and water on the day before the FDG imaging. One hour before the scan, ketamine (30 mg/kg, i.p.) and [^18^F]FDG (400 μCi, intravenous injection) were administered. An intravenous injection of [^18^F]FDG of 6.67 μCi per gram of body weight was given. After injection, the mice were maintained under anesthesia with isoflurane gas (2%–3% with 100% O_2_ at 1 L/min) during the 1-h uptake period and throughout the imaging session. FDG imaging were acquired for 10 min. The acquired FDG images were analyzed using PMOD software (PMOD Technologies Ltd.), and the standardized uptake value (SUV) was measured by defining volumes of interest (VOIs) in the whole brain.

### Standardized uptake value

The standardized uptake value (SUV), a semi-quantitative measure derived from [^18^F]FDG PET imaging, reflects the level of glucose uptake in tissue and is widely used to assess metabolic activity in various brain regions under physiological and pathological conditions (Mosconi, 2013). The standardized uptake value (SUV) was determined using the following formula:

$$SUV=\frac{Tissue\;Radioactivity\;concentration\;\left(\frac{kBq}{mL}\right)}{\frac{Injected\;Dose\;\left(MBq\right)}{Body\;Weight\;\left(kg\right)}}$$

Tissue radioactivity concentration represents the amount of FDG uptake within a defined volume, measured in kBq/mL. This value was obtained from PET images using a volume of interest (VOI), delineated in the PMOD image analysis software (PMOD Technologies LLC, Zurich, Switzerland). The VOI was drawn to encompass the entire mouse brain across all slices, with non-brain areas manually excluded on a slice-by-slice basis to ensure accuracy. The injected dose was corrected for residual activity remaining in the syringe after administration, calculated as follows:

$$Injected\;Dose\;\left(MBq\right)=Pre\;Injected\;FDG\;activity\;\left(\mu Ci\right)-Post\;Injected\;residual\;dose\;\left(\mu Ci\right)$$

The mean SUV was derived using PMOD’s statistical analysis function, which calculates the average radioactivity concentration within the defined VOI. This approach enables standardized and reproducible quantification of FDG uptake, providing reliable insights into regional brain metabolism (Stahl et al., 2004).

### Western blot analysis

Western blot analysis was performed to assess the expression of glycolysis-related proteins. Striatal tissue samples were homogenized in ice-cold RIPA Lysis and Extraction Buffer (Thermo Fisher Scientific, MA, United States). A total of 15 μg of protein from each homogenate was subjected to 10% SDS-PAGE under reducing conditions. The proteins were then transferred to polyvinylidene fluoride (PVDF) membranes using a transfer buffer at 400 mA for 2 h at 4 °C. After transfer, membranes were blocked with 5% skim milk for 2 h at room temperature and then incubated overnight at 4 °C with primary antibodies (1:1000 dilution) against PKM2 (4053, Cell Signaling Technology, MA, United States), β-actin (sc-47778, Santa Cruz Biotechnology, TX, United States), and HK-1 (2024, Cell Signaling Technology, MA, United States). After two washes with Tween 20/Tris-buffered saline (TTBS), the membranes were incubated with horseradish peroxidase (HRP)-conjugated secondary antibodies (1:3000 dilution) for 2 h at room temperature. Following three additional washes with TTBS, protein bands were visualized using an enhanced chemiluminescence (ECL) kit (Amersham Life Science, Arlington Heights, IL, United States) and detected on AGFA medical X-ray blue film. Band intensities were quantified using NIH ImageJ software (version 1.53C), and protein expression levels were normalized to β-actin as the internal control.

### RT-PCR

RT-PCR was performed to assess changes in PKM2, GLUT1, caspase-3, GAD67, and NMDA receptor expression, providing additional molecular validation for observed metabolic alterations. Total RNA was extracted from the striatum tissue using Trizol reagent (Gibco Laboratories, Gaithersburg, MD, United States) following the manufacturer’s instructions. Complementary DNA was synthesized from 1 μg of total RNA using the TOYOBO ReverTra Ace® qPCR RT Master Mix (Toyobo, Osaka, Japan). PCR amplification was carried out with ready-2x-Go Taq (NanoHelex, Daejeon, Korea) in a C1000 Touch™ Thermal Cycler (Bio-Rad Laboratories, CA, United States), following the recommended procedure. The resulting PCR products were separated on a 1.5% agarose gel prepared with TAE buffer and subsequently visualized using the GelDoc Go Gel imaging system (Bio-Rad Laboratories, CA, United States). Gene expression levels were quantified using ImageJ software. The primer nucleotide sequences used were as seen in Table 1.

**TABLE 1:** PCR primers.

Gene	Direction	Sequence	Size (bp)
Glut1	Forward	GGG​TCT​TAA​GTG​CGT​CAG​GG	312
NM_011400.3	Reverse	AGA​GAG​ACC​AAA​GCG​TGG​TG	
PKM2	Forward	CAT​TAC​CAG​CGA​CCC​CAC​AG	102
NM_011099.3	Reverse	CTC​CTG​CCA​GAC​TTG​GTG​AG	
Caspase 3	Forward	AGC​TTG​GAA​CGG​TAC​GCT​AA	241
NM_001284409	Reverse	CGT​CCA​CAT​CCG​TAC​CAG​AG	
NR2B	Forward	CCT​CCT​GTG​TGA​GAG​GAA​AGA	322
NM_001363750	Reverse	AGG​GAA​TCT​CAG​GGT​GTG​GA	
GAD67	Forward	GTC​CAG​CGT​CTG​GTT​TGA​GA	166
NM_001312900	Reverse	GTC​CGC​GCT​TGT​TGT​CAT​AG	
β-actin	Forward	GAT​TAC​TGC​TCT​GGC​TCC​TAG	147
NM_007393.5	Reverse	ACT​CAT​CGT​ACT​CCT​GCT​TG	

### Statistical analysis

All statistical analyses were performed using GraphPad Prism (version 6.07, GraphPad Software, CA, United States) unless otherwise stated. For FDG-PET studies, standardized uptake values (SUVs) were extracted from whole-brain volumes of interest using PMOD software (version 4.4, PMOD Technologies Ltd.). Because the same animals (n = 3) were scanned longitudinally at four time points (Pre, 1H, 1W, and 1M), repeated-measures statistics were applied. Specifically, Friedman test (non-parametric repeated measures ANOVA) was used to evaluate overall temporal changes, and paired comparisons (e.g., Pre vs. 1H, 1H vs. 1M) were additionally performed using paired t-tests or Wilcoxon signed-rank tests. Given the small sample size, these analyses should be interpreted as exploratory.

For molecular assays (RT-PCR and Western blot), separate groups of animals (n = 3 per time point) were used for cross-sectional comparisons. Data were expressed as mean ± SEM and analyzed using one-way ANOVA followed by Tukey’s *post hoc* test.

Correlation analyses between gene expression levels (e.g., GLUT1, caspase-3, and NR2B) were performed using Spearman’s rank correlation, and correlation coefficients (ρ) were reported. A p-value <0.05 was considered statistically significant.

## Results

### Changes in glucose metabolism following ketamine administration

**FIGURE 2:**
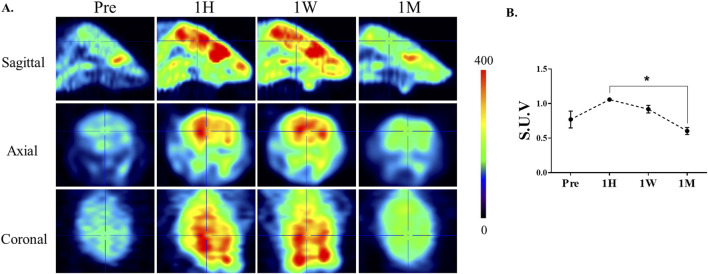
Alterations in cerebral glucose metabolism after ketamine administration. **(A)** Representative 18F-FDG PET images of mouse brains at baseline (Pre), 1 h (1H), 1 week (1W), and 1 month (1M) following chronic ketamine administration. The images illustrate an apparent increase in FDG uptake at 1 h and 1 week, followed by a reduction toward baseline levels at 1 month. Color bar indicates relative uptake intensity. **(B)** Quantitative analysis of standardized uptake values (SUVs) measured in selected brain regions, including the prefrontal cortex, hippocampus, and striatum. Data are expressed as mean ± SE (n = 3). Paired comparisons revealed a significant reduction in FDG uptake between 1H and 1M (*p < 0.05, paired t-test).

To examine the temporal effects of ketamine on cerebral glucose metabolism, FDG-PET was performed at multiple time points following ketamine administration. As shown in Figure 2, brain-wide FDG uptake increased at 1 h and remained elevated after 1 week compared to baseline. Importantly, paired analysis revealed a significant reduction between 1 h and 1 month (p = 0.013, paired t-test), indicating a biphasic metabolic response to ketamine. Notably, FDG-PET imaging revealed prominent changes in metabolic activity within the striatal region across these time points. Which guided our decision to focus subsequent molecular analyses on this area. The striatum is also a known target of ketamine’s action, implicated in reward, motivation, and dopaminergic signaling, which are often affected by chronic exposure.

### Changes in FDG uptake did not correlate with glycolytic protein and mRNA levels

**FIGURE 3:**
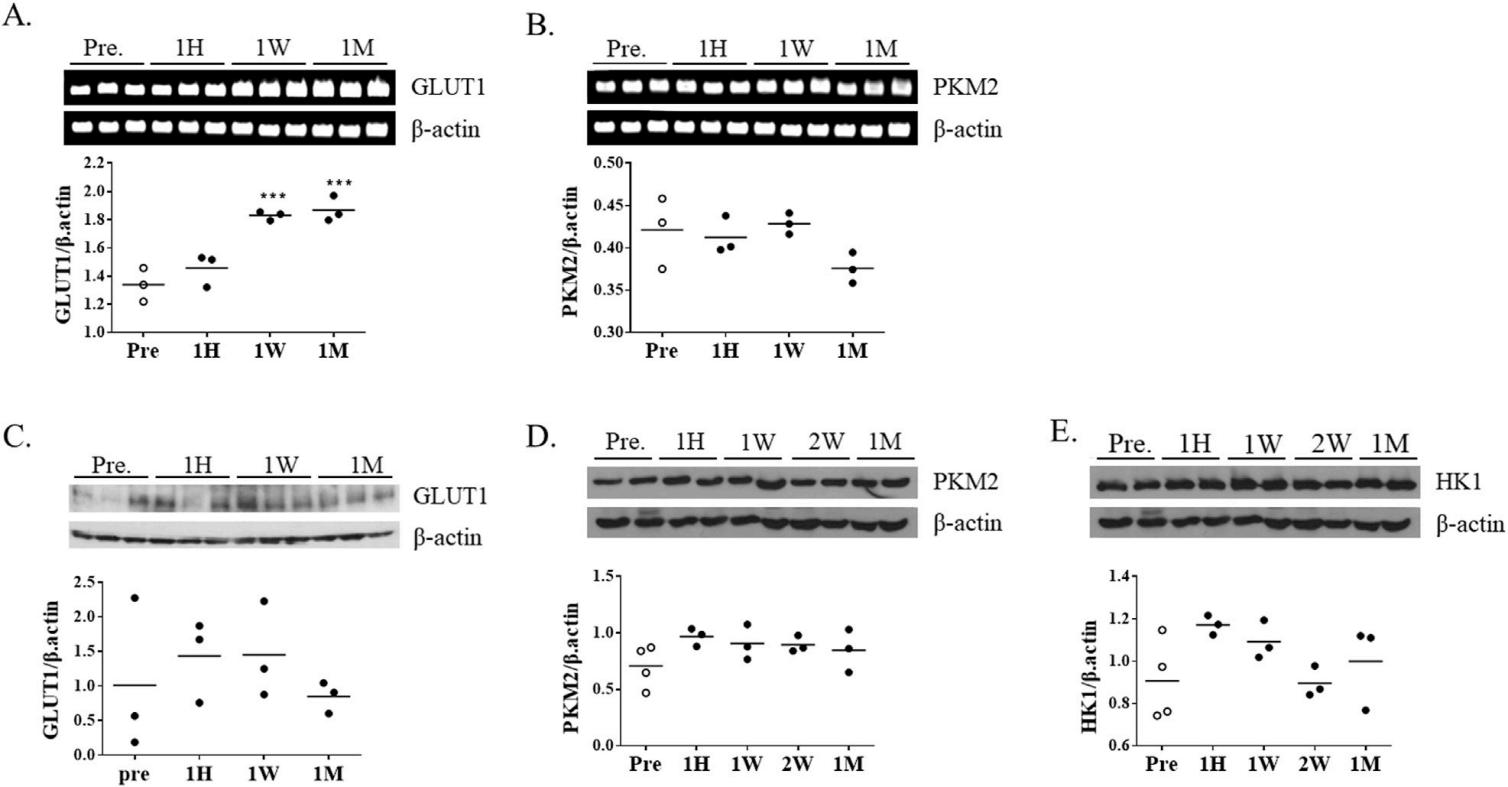
Changes in glycolysis-related gene and protein expression following chronic ketamine administration. **(A,B)** Representative RT-PCR bands and quantitative analysis of glucose transporter 1 (GLUT1) and pyruvate kinase M2 (PKM2) mRNA expression (n = 3 per group), with β-actin used as the internal control. **(C–E)** Western blot analysis of GLUT1, PKM2, and hexokinase 1 (HK1) protein expression, normalized to β-actin. Data are presented as mean ± SEM, with individual data points overlaid to indicate biological replicates (n = 3). Statistical significance was assessed by one-way ANOVA followed by Tukey’s *post hoc* test. *p < 0.05, **p < 0.01 vs. Pre-group.

To determine whether FDG uptake changes were accompanied by alterations in glycolytic gene expression, mRNA and protein levels of representative glycolytic genes were analyzed using striatal tissue. PKM2 (pyruvate kinase M2) was selected as a key glycolytic enzyme catalyzing the final step of glycolysis, while GLUT1 (glucose transporter 1) was chosen to assess glucose transport activity. RT-PCR analysis showed that GLUT1 mRNA expression gradually increased with longer exposure to ketamine (Figure 3A); however, no corresponding changes were observed at the protein level (Figure 3C). In contrast, PKM2 mRNA expression remained unchanged across all time points (Figure 3B). Consistently, the protein levels of PKM2 and HK1 (hexokinase 1), another key glycolytic enzyme, showed no significant differences between groups (Figures 3D,E).

### Long-term exposure to ketamine leads to increased caspase 3 and NR2B expression

**FIGURE 4:**
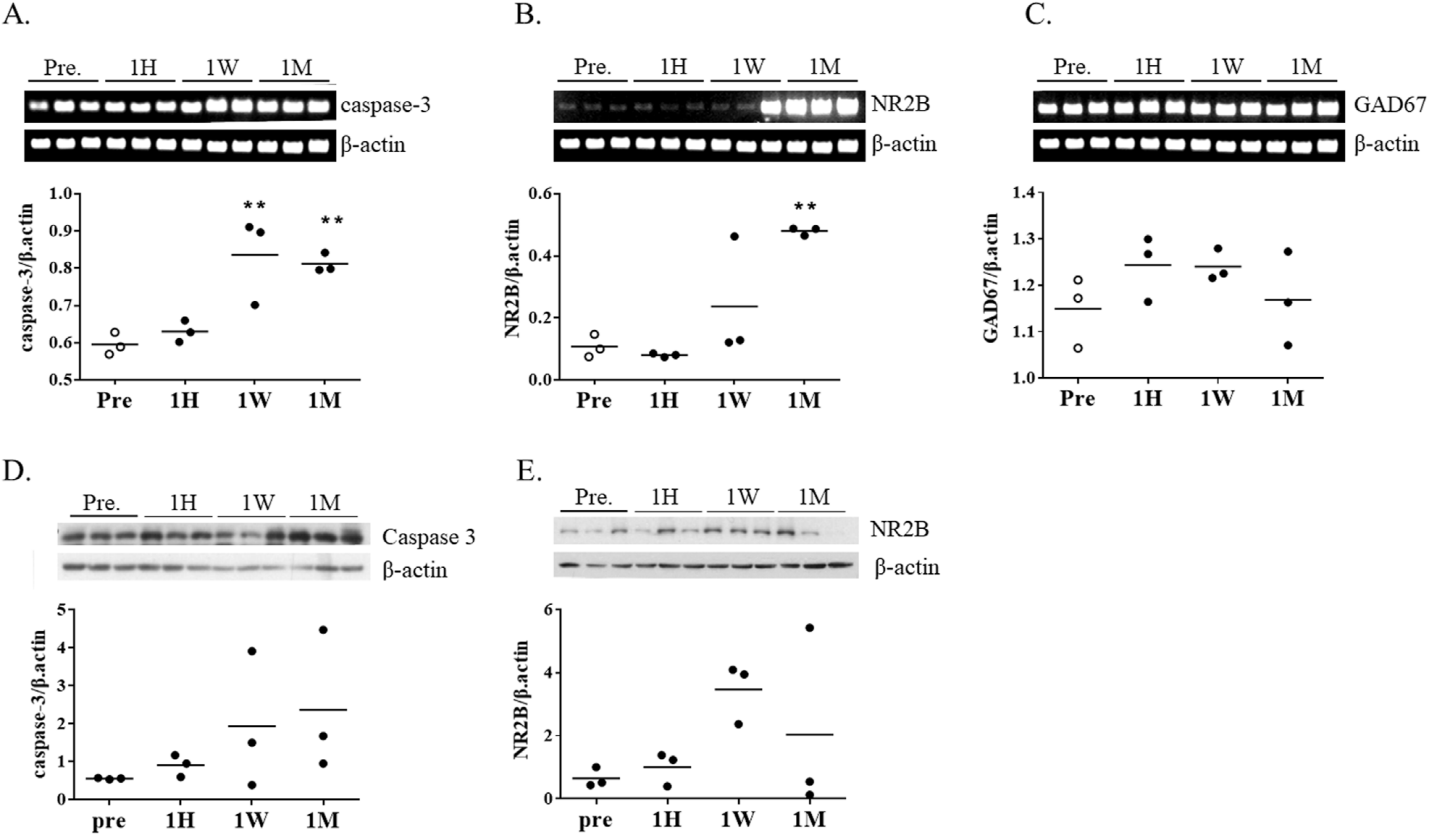
mRNA expression of apoptosis- and neurotransmission-related genes following chronic ketamine administration. **(A–C)** Relative mRNA levels of caspase-3, N-methyl-D-aspartate receptor subunit 2B (NR2B), and glutamate decarboxylase 67 (GAD67) in mouse brain tissue, normalized to β-actin (n = 3 per group) **(D,E)** Western blot analysis of caspase-3 and NR2B protein expression, normalized to β-actin. Data are presented as mean ± SEM, with individual data points overlaid to indicate biological replicates (n = 3). Statistical analysis was performed using one-way ANOVA with Tukey’s *post hoc* test. *p < 0.05, **p < 0.01, ***p < 0.001 vs. pre-group.

To investigate potential cellular responses to ketamine-induced metabolic changes, the expression of caspase-3, a central executioner of apoptosis, was examined in striatal tissue. Caspase-3 mRNA expression began to increase after 1 week of ketamine exposure and was further elevated after 1 month (Figure 4A). Consistently, caspase-3 protein levels showed a trend toward elevation at both 1 week and 1 month, although the changes did not reach statistical significance (Figure 4D).

To assess changes in excitatory and inhibitory neurotransmission, we measured expression levels of NR2B (a subunit of NMDA receptors NMDA2B, also known as GluN2B) and GAD67 (glutamate decarboxylase 67), an enzyme involved in GABA synthesis. NR2B expression tended to increase at 1 week, followed by a marked increase at 1 month (Figure 4B). However, in contrast to the transcriptional upregulation, NR2B protein levels were reduced at 1 month, primarily because two of the three animals showed lower expression, suggesting possible post-transcriptional regulation (Figure 4E). GAD67 levels remained unchanged throughout the exposure period (Figure 4C).

### Expression correlation among GLUT1, caspase-3, and NR2B expression

**FIGURE 5:**
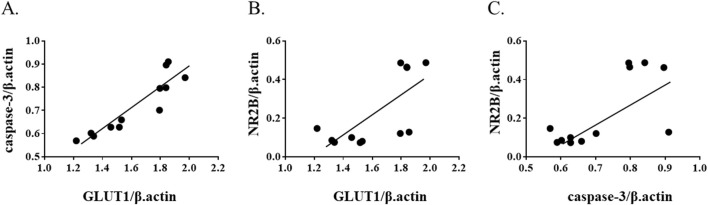
Correlation among GLUT1, cleaved caspase-3, and NR2B expression at 1 month after ketamine exposure. Scatter plots show correlations between **(A)** GLUT1 and caspase-3, **(B)** GLUT1 and NR2B, and **(C)** caspase-3 and NR2B mRNA expression levels. Spearman’s correlation analysis demonstrated a strong positive correlation between GLUT1 and caspase-3 (ρ = 0.972, p = 1.3 × 10^−7^), and moderate positive correlations between NR2B and GLUT1 (ρ = 0.622, p = 0.031) and between NR2B and caspase-3 (ρ = 0.580, p = 0.048).

To further examine molecular associations in the chronic phase of ketamine exposure, correlation analyses were performed using mRNA expression levels at the 1‐month time point. Spearman’s rank correlation revealed a strong positive correlation between GLUT1 and caspase‐3 (ρ = 0.972, p = 1.3 × 10^−7^) (Figure 5A). In addition, moderate positive correlations were observed between NR2B and GLUT1 (ρ = 0.622, p = 0.031) (Figure 5B) and between NR2B and caspase‐3 (ρ = 0.580, p = 0.048) (Figure 5C). These findings suggest coordinated regulation of glucose transport, apoptotic signaling, and NMDA receptor expression during prolonged ketamine exposure.

## Discussion

In this study, we observed dynamic, time-dependent changes in brain glucose metabolism following ketamine administration, reflecting complex neurobiological adaptations associated with chronic exposure. FDG-PET findings demonstrated a biphasic trajectory, with glucose uptake increasing at 1 h and remaining elevated at 1 week, followed by a significant decline at 1 month (1H vs. 1M, p = 0.013, paired t-test) that returned values toward baseline. This pattern suggests that ketamine’s acute excitatory effects are transient, whereas prolonged exposure engages adaptive or stress-related mechanisms that normalize cerebral metabolism. This interpretation is consistent with previous reports showing that subanesthetic ketamine acutely enhances cortical excitability and energy demand through glutamatergic disinhibition and synaptic plasticity (Grieco et al., 2020; Abdallah et al., 2016; Duman et al., 2012), whereas prolonged exposure has been associated with disrupted neuronal homeostasis, oxidative stress, and impaired mitochondrial function (Ito et al., 2015). The prominent striatal changes observed across these time points further support its role as a key region mediating ketamine’s neuropharmacological effects, consistent with its involvement in reward, motivation, and dopaminergic signaling.

Despite the decline in FDG uptake during the chronic phase, GLUT1 mRNA expression gradually increased, while protein levels remained unchanged. This dissociation may suggest that transcriptional upregulation of GLUT1 does not directly translate into enhanced glucose utilization, but could represent a compensatory response to diminished intracellular glucose availability. Similar GLUT1 induction has been reported under hypoxic, inflammatory, or hypoglycemic stress (Kumagai et al., 1995). In this context, the transcript changes observed here may reflect an adaptive attempt to sustain cerebral glucose access under metabolic stress imposed by prolonged ketamine exposure. Activation of immune responses is typically accompanied by a metabolic shift toward glycolysis (O’Neill et al., 2016). Although inflammatory mediators were not directly assessed, ketamine has been reported to modulate neuroimmune responses, including suppression of pro-inflammatory cytokines and normalization of glial activation (Valenza et al., 2024). Nevertheless, the metabolic pattern observed in this study does not align with a classical immune-driven glycolytic shift. FDG uptake changes and the selective upregulation of GLUT1, in the absence of PKM2 or HK1 induction, suggest that the adaptations we observed are more consistent with neuronal compensatory responses rather than inflammation-associated glycolysis. Immune-related pathways may still exert indirect effects, but are unlikely to represent the primary driver of the metabolic phenotype detected by FDG-PET.

Our data are consistent with the possibility that prolonged ketamine exposure induces metabolic stress that may not be fully compensated by glucose transporter upregulation, potentially resulting in a mismatch between energy supply and neuronal demand, although further validation is required. This stress may interact with synaptic remodeling, as indicated by NR2B dysregulation, and with early apoptotic signaling, as suggested by caspase-3 expression. These findings raise the possibility that metabolic, synaptic, and cell death pathways are interrelated during chronic ketamine exposure, although further validation with larger sample sizes will be required.

A particularly noteworthy observation was the dissociation between NR2B transcript and protein levels. While NR2B mRNA expression increased markedly at 1 month, protein levels did not show a corresponding rise, with some animals even exhibiting reductions. Such transcript–protein mismatch has been reported for neurotransmitter receptors due to post-transcriptional regulation, trafficking, or degradation (Follesa and Ticku, 1996; Gallager et al., 1984; Matsumoto et al., 1996). Importantly, similar discrepancies have been linked to impaired synaptic plasticity and neuropsychiatric disorders (Paoletti et al., 2013). In our study, this mismatch may reflect compensatory mechanisms that fail to restore protein-level function. This could be related to the attenuated FDG response observed at the same time point, although this interpretation remains speculative.

Correlation analyses provided additional insight, albeit limited to transcript levels. Strong positive correlations were observed between GLUT1 and caspase-3, and moderate correlations between NR2B and both GLUT1 and caspase-3. These associations raise the possibility of coordinated regulation of glucose transport, apoptotic signaling, and NMDA receptor remodeling during chronic ketamine exposure. However, given the small sample size and reliance on mRNA data, these results should be regarded as exploratory.

In contrast to excitatory pathways, inhibitory signaling appeared relatively stable, as GAD67 expression did not change across time points. While this may suggest preserved GABAergic tone, more refined region-specific or single-cell analyses will be required to detect subtler changes not captured in bulk assays or FDG-PET (Li et al., 2017).

Although our protocol employed higher doses than those typically used in depression models, it is noteworthy that even therapeutic ketamine regimens can exhibit tolerance, diminished efficacy, or abuse potential over time. Prior work has shown that ketamine restores cortical glucose uptake via the ERK–GLUT3 pathway in depression models (Ouyang et al., 2021). While we did not assess GLUT3, our data similarly revealed an early metabolic enhancement followed by normalization, consistent with waning responsiveness during prolonged exposure. Thus, while our findings cannot establish a direct causal link, they are compatible with the idea that ketamine induces both adaptive and maladaptive, time-dependent alterations in brain metabolism. The balance between these opposing effects likely depends on dose, duration, and individual neurobiology.

Several limitations be acknowledged. First, the small sample size represents a major limitation of this study. Because only three animals completed all serial FDG-PET scans, paired statistical analyses were restricted to these, which reduces statistical power and limits the generalizability of the findings (Button et al., 2013). Thus, the present data should be regarded as exploratory, although the consistent biphasic pattern observed across serially imaged animals provides biological plausibility and is in line with prior reports (Grieco et al., 2020; Abdallah et al., 2016; Duman et al., 2012; Ito et al., 2015). Second, the limited number of imaging time points constrains the ability to capture finer-grained temporal dynamics of ketamine’s effects. Third, the absence of behavioral or cognitive assessments represents a notable limitation, as it prevents direct linkage between metabolic changes and functional outcomes. Lastly, FDG-PET measures glucose uptake but not glycolytic flux or ATP production. To enhance mechanistic understanding, future studies should integrate additional analyses such as lactate quantification, assessments of mitochondrial respiratory function, and evaluation of oxidative phosphorylation efficiency.

Overall, our findings highlight ketamine’s biphasic impact on cerebral metabolism: early hypermetabolism followed by normalization and engagement of apoptotic and receptor remodeling pathways. While the molecular data should be interpreted with caution due to small sample size and transcript–protein discrepancies, the imaging results provide the central evidence of this study. These findings underscore the potential of metabolic imaging as a non-invasive tool to monitor ketamine’s temporal effects. Techniques such as ultra-low-dose FDG-PET and hyperpolarized ^13^C-MRS may offer valuable biomarkers to guide safe and personalized use of ketamine in neuropsychiatric conditions (Morris and Bachelard, 2003; Choi et al., 2018).

## Conclusion

In conclusion, this study suggests that ketamine exposure may induce temporally distinct alterations in brain glucose metabolism, characterized by an apparent early hypermetabolic phase followed by a return toward baseline by 1 month. These metabolic changes were accompanied by molecular adaptations, although transcript–protein discrepancies indicate the need for cautious interpretation. Together, these findings raise the possibility that ketamine triggers both adaptive and stress-related responses in an exposure-dependent manner. While additional studies with larger sample sizes and behavioral correlations are warranted, our results suggest that metabolic imaging may serve as a non-invasive tool to track the temporal dynamics of ketamine’s effects and to aid in evaluating its long-term safety. Such insights may help guide the safer application of ketamine in clinical practice.

## Data Availability

All data that support the findings of this study are available from the corresponding author upon reasonable request.
